# The Safety and Efficacy of Ceftazidime-Avibactam Plus Metronidazole Versus Meropenem for Intra-abdominal Infections: A Systematic Review and Meta-Analysis

**DOI:** 10.7759/cureus.86262

**Published:** 2025-06-18

**Authors:** Mahmoud Ismail Nouh, Hala AlButi, Hani Raka Karrar, Dina M Hassan, Rehab Salah Aldin Alhendi, Hanin Ali Aseeri, Mohannad A Algothmi, Abdulrahman Olayan Mohammed Almuqati, Khloud Mubark Alotaibi, Nouran M Alkhaifi, Samar Y Badayyan, Lamer K Shaikh, Nourah A Al Ghamdi, Fai F Abdullah

**Affiliations:** 1 Research and Continuing Education Committee, Pharmaceutical Care Department, King Fahad Armed Forces Hospital, Jeddah, SAU; 2 Pharmaceutical Care Department, King Fahad Armed Forces Hospital, Jeddah, SAU; 3 Pharmaceutical Care Department, Dr. Samir Abbas Hospital, Jeddah, SAU; 4 Laboratory Department, Dr. Samir Abbas Hospital, Jeddah, SAU; 5 Obstetrics and Gynecology Department, Ministry of Health, Makkah, SAU; 6 Medicine and Surgery, Ibn Sina National College, Jeddah, SAU; 7 Medicine, King AbdulAziz University, Jeddah, SAU; 8 Therapeutics, Shaqra General Hospital, Shaqra, SAU; 9 Pharmaceutical Care Department, Security Forces Hospital, Makkah, SAU; 10 Medicine and Surgery, Fakeeh College for Medical Sciences, Jeddah, SAU

**Keywords:** avibactam, ceftazidime, intra abdominal infections, meropenem, metronidazole

## Abstract

Intra-abdominal infections encompass a range of medical conditions categorized by their complexity. Uncomplicated infections involve inflammation or infection limited to a single abdominal organ, such as acute appendicitis or cholecystitis, without extending to the peritoneum, while complicated infections spread to the peritoneal cavity. The key associated microbiological agents include Gram-positive cocci, Gram-negative Enterobacteriaceae, and obligate anaerobes, with common pathogens being Escherichia coli, Klebsiella pneumoniae, Streptococcus species, and Bacteroides fragilis. Treatment options include well-established antibiotics and newer agents like meropenem, metronidazole, and ceftazidime/avibactam. Meropenem, a carbapenem antibiotic, is known for its broad-spectrum efficacy and low toxicity, making it suitable for severe infections. Ceftazidime, a third-generation cephalosporin, is effective against Pseudomonas aeruginosa, especially when paired with avibactam, a β-lactamase inhibitor, enhancing its effectiveness. Metronidazole disrupts bacterial DNA, targeting anaerobic bacteria and protozoa.

This systematic review and meta-analysis aimed to evaluate the safety and efficacy of the ceftazidime-avibactam plus metronidazole combination compared to meropenem for intra-abdominal infections. Following the Preferred Reporting Items for Systematic Review and Meta-Analysis (PRISMA) guidelines, a comprehensive search of databases such as PubMed, Web of Science, and the Cochrane Library was conducted. Results showed that the combination therapy had a slightly higher overall adverse event rate (5.57%) compared to meropenem (4.56%), although this difference was not statistically significant [risk ratio (RR): 1.22; 95% confidence interval (CI): 0.78-1.93; p = 0.39]. Meropenem demonstrated a significantly higher clinical response rate in ceftazidime-susceptible infections (89.93% vs. 85.88%; RR: 0.96; 95% CI: 0.93-0.99; p = 0.009). No significant differences were observed in ceftazidime-resistant infections. Overall, the findings suggest that ceftazidime-avibactam combined with metronidazole is a viable alternative to meropenem, highlighting the need for further research to optimize treatment strategies amid rising antibiotic resistance.

## Introduction and background

Intra-abdominal infections encompass a variety of medical conditions, and they can be categorized based on their complexity. Uncomplicated intra-abdominal infections occur when there is an infection or inflammation in the wall of an abdominal organ, like acute appendicitis or cholecystitis. These types of infections affect just one organ and do not spread to the peritoneum, meaning there is no disruption in the gastrointestinal system. On the other hand, complicated intra-abdominal infections happen when the infection spreads into the peritoneal area, making the condition more serious [1,2]. Depending on the infection's origin, the patient's underlying physiological reserves, and the effectiveness of previous therapy, the prognosis for these infections varies significantly. Patient outcomes have improved as a result of improved antibiotic efficacy, accessible CT imaging for early diagnosis, and advanced interventional radiology treatment approaches [3,4].

Gram-positive cocci and Gram-negative Enterobacteriaceae, as well as obligate anaerobes, are the main microbiological causes of intra-abdominal infections. Most of these microorganisms are transmitted via intra-abdominal fluid and tissue cultures, including Escherichia coli, Klebsiella pneumoniae, Streptococci, and Bacteroides fragilis. As there is a large range of bacteria that might cause intra-abdominal infections, broad-spectrum antimicrobial medicines are used as an empirical therapy [5,6]. Intra-abdominal infections can be treated with a variety of antibiotic medicines. Several drugs, including meropenem, metronidazole, and ceftazidime/avibactam, have also been licensed for the treatment of intra-abdominal infections, in addition to more established antimicrobials. The primary objective of this study was to compare meropenem and ceftazidime/avibactam plus metronidazole [7, 8].

Meropenem is a β-lactam antibiotic belonging to the carbapenem family, and it has low toxicity and a wide spectrum of action. This antibiotic is effective and frequently used for the treatment of severe and nosocomial infections in hospitalized patients because it offers protection against a variety of bacteria [9,10]. Meropenem has a time-dependent bactericidal activity that prevents the formation of bacterial cell walls [11]. The primary indicator of treatment efficacy for medications that rely on time is the proportion of time that the infection site's antibiotic levels are above the pathogen's minimum inhibitory concentration. Therefore, the likelihood of improving clinical therapeutic success increases with the length of time blood concentrations stay above the minimum inhibitory concentration [12,13].

Ceftazidime is a third-generation cephalosporin active against Pseudomonas aeruginosa and was often used as monotherapy in the 1990s. The indications of extended-spectrum β-lactamases were gradually limited to the specific treatment of Pseudomonas aeruginosa infections. Avibactam functions as a class A β-lactamase inhibitor. Unlike clavulanic acid and tazobactam, avibactam does not hydrolyze upon forming a covalent connection with the serine of the β-lactamase active center. Instead, it slowly separates and reassembles into its original structure. The observed increased efficacy can be explained by this method of action: a broad range of activity against various β-lactamases, and an elimination half-life of 2.5 hours. Ceftazidime avibactam has an antibacterial spectrum that includes >99% of enterobacteria and 95% of isolates of Pseudomonas aeruginosa [14-17].

Metronidazole is frequently prescribed to treat a variety of infectious disorders. Nitroimidazole refers to the family of drugs to which metronidazole belongs. It functions by destroying the DNA of bacterial cells. Metronidazole is regarded as the most commonly used medicine in the nitroimidazole class. Nitroimidazoles are prodrugs activated in low-oxygen environments. This is accomplished by reducing the nitro group, which produces imidazole and causes cytotoxicity. However, metronidazole's cytotoxicity and metabolic mechanism are still not well understood [18-21]. Metronidazole has a potent antibacterial action with excellent coverage that is not limited to the inhibition of microorganisms. Additionally, it can eliminate specific types of other organisms, including worms and protozoa. In 1959, metronidazole was introduced to the market as a successful treatment for Trichomonas vaginalis. When it comes to Gram-positive anaerobic bacteria like Peptostreptococci spp., metronidazole offers excellent coverage. Additionally, it provides adequate coverage for anaerobic Gram-negative bacteria, including Bacteroides species and Fusobacterium. Metronidazole can attack various types of protozoa such as Trichomonas vaginalis, Entamoeba histolytica, and Giardia lamblia [22-26].

However, there is a lack of a systematic analysis regarding the use of ceftazidime-avibactam plus metronidazole versus meropenem in intra-abdominal infections. Therefore, we conducted a systematic review to assess the safety and efficacy of ceftazidime-avibactam plus metronidazole versus meropenem in intra-abdominal infections.

## Review

Materials and methods

This systematic review employed the Preferred Reporting of Systematic Reviews and Meta-Analyses (PRISMA) 2020 standards were followed in [27].

Search Strategy and Selection Criteria

We used the search terms "Ceftazidime," "Avibactam," "Metronidazole," "Meropenem," and "Intra-Abdominal Infections." Articles were found using the bibliographic databases of MEDLINE (via PubMed), Web of Science, and the Cochrane Library. The search was limited to randomized controlled trial studies. No restrictions were placed on language. Two authors (N. Alkhaifi and M. Nouh) carried out the searches and data extractions separately until Jan 22, 2024. The PICOS criteria served as the foundation for this study's inclusion requirements [28-29]. 1) Population: Any patients who have intra-abdominal infections (complicated or uncomplicated). 2) Intervention/Comparison: ceftazidime-avibactam plus metronidazole/meropenem. 3) Outcomes: The efficacy, which includes the clinical response at the test-of-cure visit, and the safety, which encompasses the incidence rate of side effects. 4) Study design: randomized controlled trial. Duplicate studies and case reports were excluded from the study.

Outcomes, Data Analysis, and Risk of Bias

The main result was safety and efficacy related to the use of ceftazidime-avibactam plus metronidazole versus meropenem. The incidence of gastrointestinal, renal, respiratory, and neurological adverse effects associated with ceftazidime-avibactam plus metronidazole versus meropenem will be assessed. Using random-effects models, Mantel-Haenszel risk ratios (RRs) with 95% confidence intervals (CIs) were evaluated. p<0.05 was a significant difference. The proportion of variation in outcomes across research is displayed in this study using the Mantel-Haenszel technique and the heterogeneity test (I^2^). Using the heterogeneity statistics, we calculated the degree of heterogeneity among the trial outcomes (25%, 50%, and 75% representing low, moderate, and high heterogeneity, respectively). Sensitivity analysis and subgroup analysis were done to look at possible sources of heterogeneity. The Cochrane Collaboration's technique for evaluating the risk of bias was used to evaluate the quality of individual studies. The software Review Manager (RevMan) Version 5.4 was used to carry out each of these tasks. The Cochrane Library developed RevMan, a program for creating and managing reviews that incorporate study data, language, comparison tables, and research characteristics. It might do a meta-analysis on the given data and present the results graphically [29-30].

Data Extraction and Sensitivity Test

Following an evaluation of the eligible studies, the following information was gathered: (1) author name; (2) year of publication; (3) study location; (4) study design; (5) the title of the study; (6) number of patients; and (7) length of course. Two reviewers (L. Shaikh and F. Abdullah) carried out the data extraction independently. Conflicts were settled by H. Karrar, the third reviewer. We used a jackknife approach to undertake a sensitivity analysis and examine the robustness of the pooled HR with 95% CI. This allowed us to assess the impact of each study on the combined effect estimate. The same method was also used to address any notable heterogeneity that may have existed [29].

Results

Results of Literature Search

**Figure 1:**
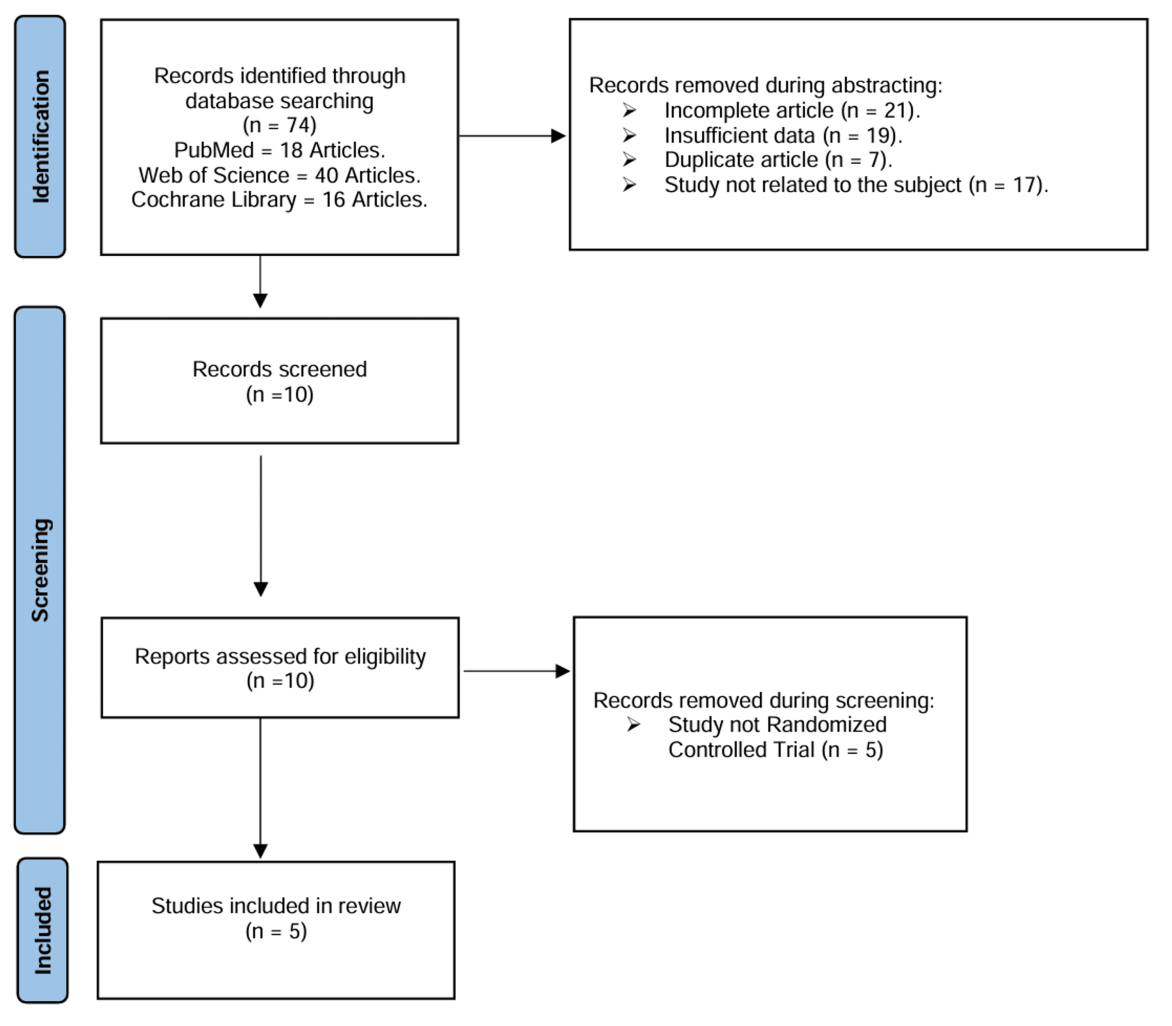
PRISMA flowchart depicting the study selection process PRISMA: Preferred Reporting Items for Systematic Reviews and Meta-Analyses

The initial literature search, which was done utilizing electronic web resources, yielded 74 relevant research studies. Ten papers were reviewed for possible inclusion, after duplicates, incomplete articles, and insufficient data were excluded based on titles and abstracts. There were still five studies available for quantitative analysis. The results and summary of the literature search are illustrated in Figure 1.

Quality of the Included Studies

**Figure 2:**
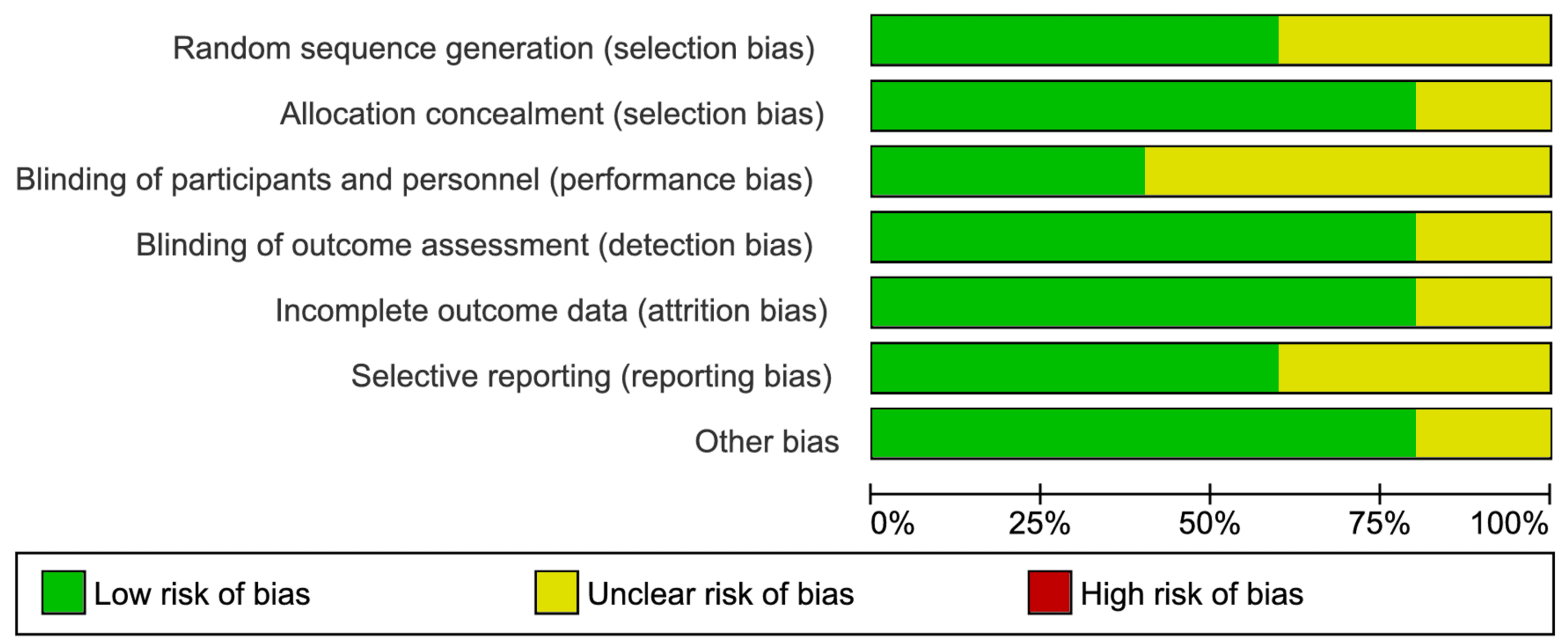
Risk of bias graph of included studies* ^*^[31-35]

**Figure 3:**
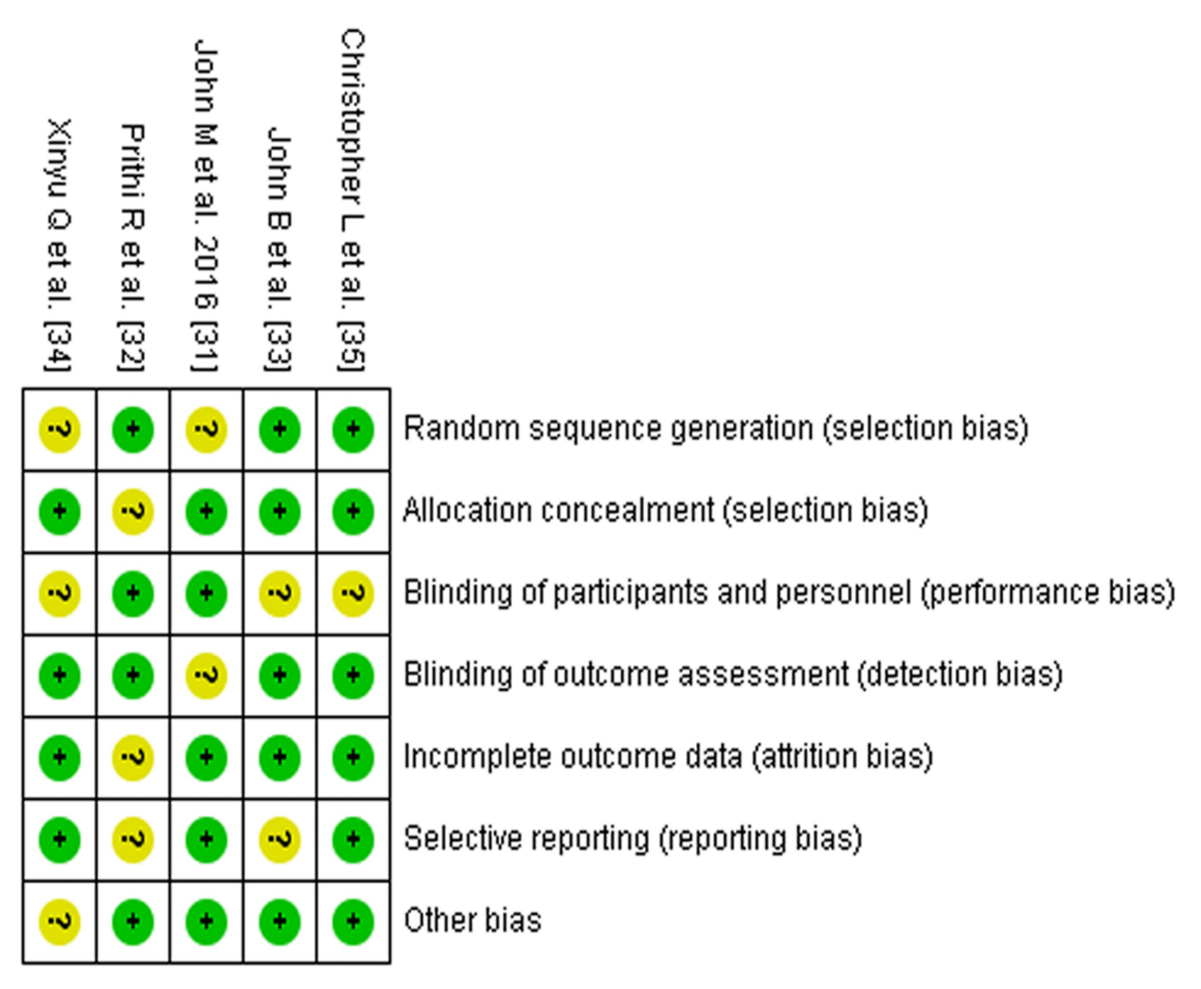
Risk of bias summary of included studies* ^*^[31-35]

The Cochrane Collaboration tool indicated that the quality of the included trials varied from low to moderate. Figure 2 displays the risk of bias graph for the quality assessment of the included studies, while Figure 3 provides a summary of the quality assessment results for the included studies.

Characteristics of the Included Studies

Our analysis included 1,890 patients, with 961 receiving ceftazidime/avibactam plus metronidazole and 929 receiving meropenem. Trials were conducted from 2013 to 2022 in the USA (n = 2), India (n = 2), Asia (n = 1), and the UK (n = 1). Treatment duration ranged from 1 to 2 weeks. Table 1 summarizes five randomized controlled trials investigating the effectiveness and safety of combining ceftazidime-avibactam with metronidazole compared to meropenem for treating intra-abdominal infections. Mazuski et al.'s [31] phase 3 study in India included 1,058 patients and lasted about one week, focusing on the efficacy of the combination therapy. Rodgers et al.'s [32] multicenter trial, also in India, involved 115 patients and assessed the combination against meropenem over approximately two weeks. Bradley et al.'s [33] phase 2 trial examined 83 patients in the UK and USA, lasting about two weeks, to evaluate safety and efficacy. Qin et al.'s [34] double-blind phase 3 trial, conducted across Asia with 431 patients, compared the combination therapy to meropenem over roughly one week. Finally, Lucasti et al.'s [35] phase 2 trial in the USA included 203 adult patients and lasted about one week, focusing on intra-abdominal infections. Collectively, these studies provide valuable insights into the safety and effectiveness of using ceftazidime-avibactam with metronidazole versus meropenem in various patient populations and clinical settings.

**Table 1:** Characteristics of included studies

Author and year of publication	Study design	Title	Location	Number of patients	Course duration
Mazuski et al., 2016 [31]	RCT: randomized, controlled, double-blind, phase 3	Efficacy and safety of ceftazidime-avibactam plus metronidazole versus meropenem in the treatment of complicated intra-abdominal infection: results from a randomized, controlled, double-blind, phase 3 program	India	1,058	Around 1 week
Rodgers et al., 2022 [32]	RCT: randomized, multicenter, double-dummy, double-blind	Ceftazidime-avibactam plus metronidazole vs. meropenem in complicated intra-abdominal infections: Indian subset from RECLAIM	India	115	Around 2 weeks
Bradley et al., 2019 [33]	RCT: phase 2, randomized, controlled trial	Safety and efficacy of ceftazidime-avibactam plus metronidazole in the treatment of children ≥3 months to <18 years with complicated intra-abdominal infection: results from a phase 2, randomized, controlled trial	UK, USA	83	Around 2 weeks
Qin et al., 2017 [34]	RCT: randomized, double-blind, phase III trial	A randomised, double blind, phase 3 study comparing the efficacy and safety of ceftazidime/avibactam plus metronidazole versus meropenem for complicated intra-abdominal infections in hospitalized adults in Asia	ASIA	431	Around 1 week
Lucasti et al., 2013 [35]	RCT: randomized, double-blind, phase II trial	Comparative study of the efficacy and safety of ceftazidime/avibactam plus metronidazole versus meropenem in the treatment of complicated intra-abdominal infections in hospitalized adults: results of a randomized, double-blind, phase II trial	USA	203	Around 1 week

Patient Demographics and Baseline Characteristics

The minimum age of the cohort was 10 years, while the maximum was 50 years. Among these patients, 64.6% were male, while 35.4% were female. The mean BMI across the studies was 22.95 kg/m^2^. Additionally, a significant proportion of the studies involved patients who had received prior antibiotic therapy. Table 2 presents patient demographics and baseline characteristics from five studies comparing ceftazidime-avibactam plus metronidazole with meropenem. Mazuski et al.'s [31] study involved 1,058 patients with a mean age of 50 years, comprising 673 males and 385 females, and a mean BMI of 26.25 kg/m²; notably, 649 patients had prior antibiotic therapy. Rodgers et al.'s [32] study included 115 patients with a mean age of 34.75 years, consisting of 56 males and 59 females, although the mean BMI was not specified, with prior antibiotic therapy noted in 50 patients. Bradley et al.'s [33] study involved 83 patients with a mean age of 10.25 years, showing a gender distribution of 53 males and 30 females and a mean BMI of 18.25 kg/m², and 74 patients had prior antibiotic therapy. Qin et al.'s [34] study involved 431 patients with a mean age of 48.5 years, including 294 males and 137 females, a mean BMI of 22.55 kg/m², and prior antibiotic therapy in 53 patients. Lastly, Lucasti et al.'s [35] study included 203 patients with a mean age of 42.8 years, with 151 males and 52 females, and a mean BMI of 24.75 kg/m², where 107 patients had received prior antibiotic therapy. These studies provide a diverse overview of the patient populations involved in evaluating the efficacy and safety of the antibiotic treatments.

**Table 2:** Patient demographics and baseline characteristics CAZ/AVI: ceftazidime/avibactam; MERO: meropenem; MET: metronidazole; SD: standard deviation

Variables	Mazuski et al., 2016 [31]		Rodgers et al., 2022 [32]		Bradley et al., 2019 [33]		Qin et al., 2017 [34]		Lucasti et al., 2013 [35]	
	CAZ/AVI + MET	MERO	CAZ/AVI + MET	MERO	CAZ/AVI + MET	MERO	CAZ/AVI + MET	MERO	CAZ/AVI + MET	MERO
No. of patients	529	529	56	59	61	22	214	217	101	102
Age, years, mean ± SD	49.8 ± 17.5	50.3 ± 18.3	34.5	35	10.4	10.1	48.5 ± 16.8	48.5 ± 17.4	43.0 ± 15.9	42.6 ± 18.1
Gender, male, n	335	338	39	17	44	9	141	153	70	81
Gender, female, n	194	191	17	42	17	13	73	64	31	21
Mean body mass index, kg/m^2^	26.3	26.2	-	-	18.1	18.4	22.7	22.4	24.2	25.3
Prior antibiotic therapy, n	324	325	23	27	54	20	26	27	53	54

Safety Profile

**Figure 4:**
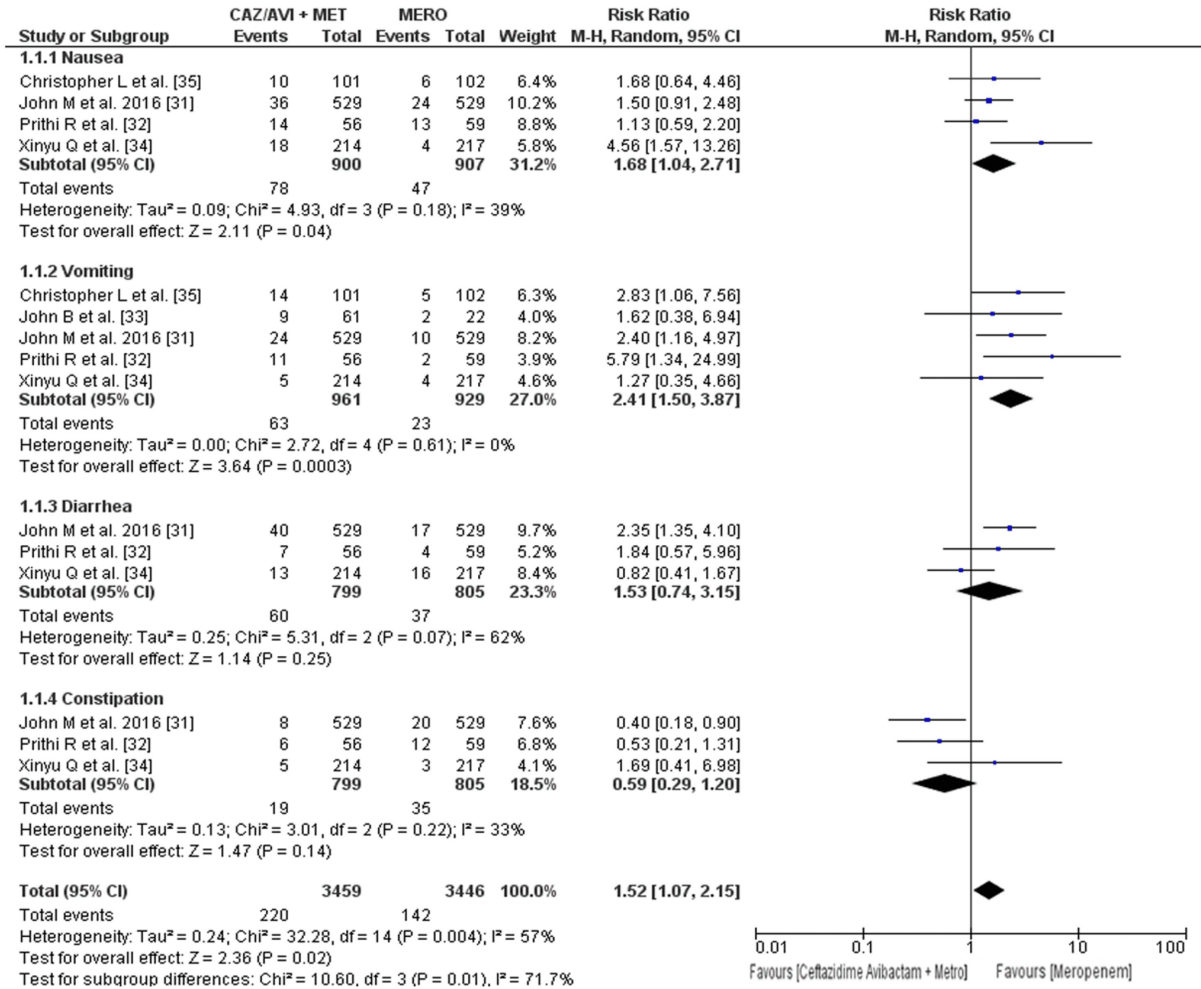
Forest plot showing the risk ratios of the incidence rate of gastrointestinal side effects using random-effects models The horizontal line represents 95% CI; a vertical line represents a “no difference” point between the two groups; squares are risk ratios; diamonds are pooled risk ratios CI: confidence interval; CAZ/AVI: ceftazidime/avibactam; MERO: meropenem; MET: metronidazole; MH: Mantel-Haenszel

The evaluation of the safety profile focused on gastrointestinal side effects, including nausea, vomiting, diarrhea, and constipation, as well as respiratory, renal, and central nervous system side effects (Figure 4). The incidence rate of nausea in patients who received ceftazidime-avibactam plus metronidazole was 8.66%, while the incidence rate in the meropenem arm was 5.18%; it was significantly higher in the ceftazidime-avibactam plus metronidazole arm (RR: 1.68; 95% CI: 1.04 to 2.71; p = 0.04). The incidence rate of vomiting in patients who received ceftazidime-avibactam plus metronidazole was 6.55%, while the incidence rate in the meropenem arm was 2.47%; it was significantly higher in the ceftazidime-avibactam plus metronidazole arm (RR: 2.41; 95% CI: 1.50 to 3.87; p = 0.0003). The incidence rate of diarrhea in patients who received ceftazidime-avibactam plus metronidazole was 7.50%, while the incidence rate in the meropenem arm was 4.59%; it was higher in the ceftazidime-avibactam plus metronidazole arm, but not significant (RR: 1.53; 95% CI: 0.74 to 3.15; p = 0.25). The incidence rate of constipation in patients who received ceftazidime-avibactam plus metronidazole was 2.37%, while the incidence rate in the meropenem arm was 4.37%; it was higher in the meropenem arm but not significant (RR: 0.59; 95% CI: 0.29 to 1.20; p = 0.14).

The overall incidence rate of GI side effects in patients who received ceftazidime-avibactam plus metronidazole was 6.36%, while the incidence rate in the meropenem arm was 4.12%, and it was significantly higher in the ceftazidime-avibactam plus metronidazole arm, as shown in Figure 4 (RR: 1.52; 95% CI: 1.07 to 2.15; p = 0.02) [29]. 

**Figure 5:**
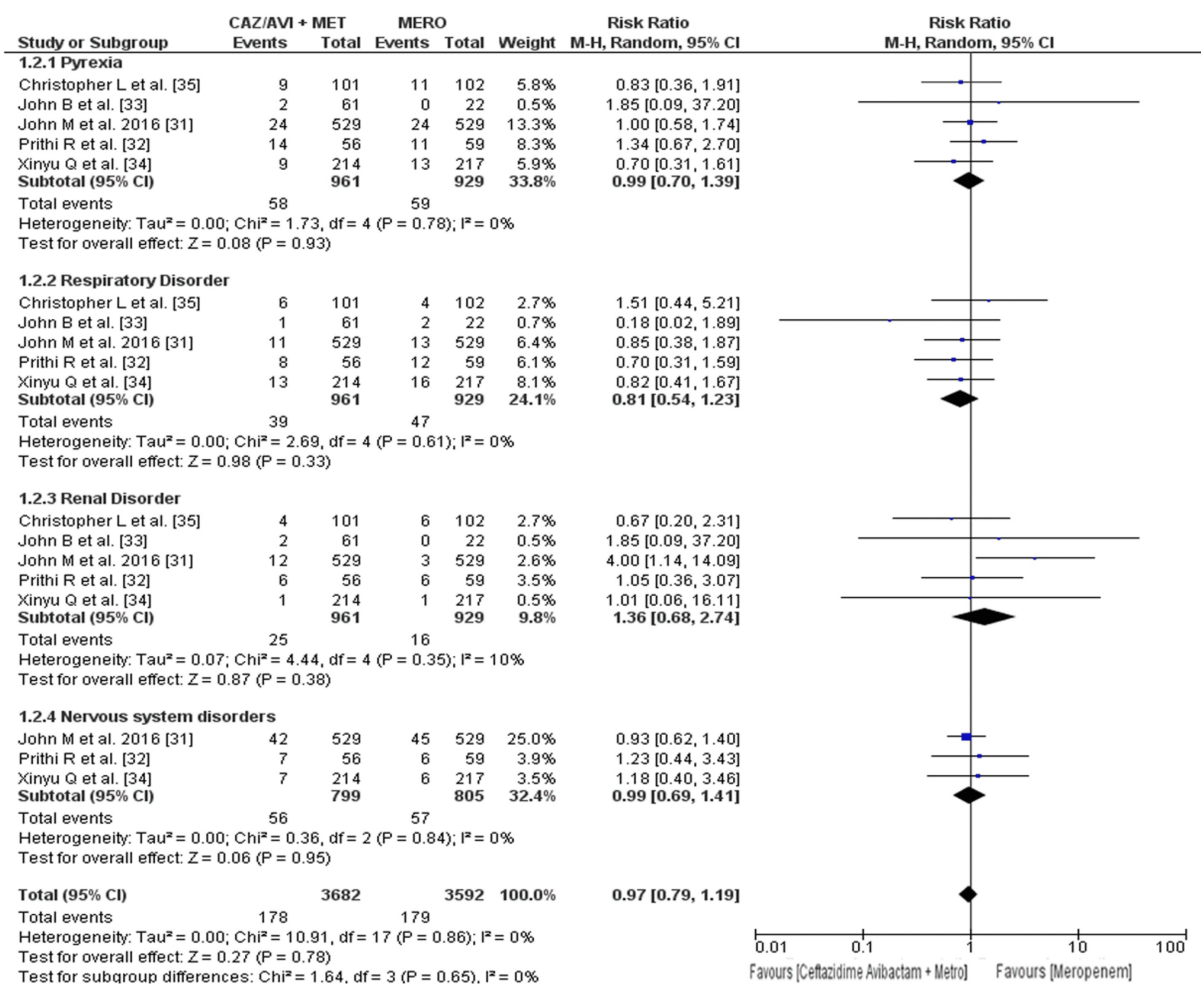
Forest plot showing the risk ratios of the incidence rate of general side effect using random-effects models The horizontal line represents 95% CI; a vertical line represents a “no difference” point between the two groups; squares are risk ratios; diamonds are pooled risk ratios. Respiratory disorders include cough, dyspnea, and lung consolidation. Renal disorders include hematuria, dysuria, renal colic, urethral meatus stenosis, and urinary retention. Nervous system disorders include headache, hypertension, hypotension, and dizziness CI: confidence interval; CAZ/AVI: ceftazidime/avibactam; MERO: meropenem; MET: metronidazole; MH: Mantel-Haenszel

The incidence rate of pyrexia in patients who received ceftazidime-avibactam plus metronidazole was 6.03%, while the incidence rate in the meropenem arm was 6.35%; it was higher in the meropenem arm but not significant (RR: 0.99; 95% CI: 0.70 to 1.39; p = 0.93). The incidence rate of respiratory disorder in patients who received ceftazidime-avibactam plus metronidazole was 4.05%, while the incidence rate in the meropenem arm was 5.05%; it was higher in the meropenem arm, but not significant (RR: 0.81; 95% CI: 0.54 to 1.23; p = 0.33). The incidence rate of renal disorder in patients who received ceftazidime-avibactam plus metronidazole was 2.60%, while the incidence rate in the meropenem arm was 1.72%; it was higher in the ceftazidime-avibactam plus metronidazole arm, but not significant (RR: 1.36; 95% CI: 0.68 to 2.74; p = 0.38). The incidence rate of nervous system disorder in patients who received ceftazidime-avibactam plus metronidazole was 7.00%, while the incidence rate in the meropenem arm was 7.08%; it was higher in the meropenem arm but not significant (RR: 0.99; 95% CI: 0.69 to 1.41; p = 0.95) (Figure 5). 

The overall incidence rate of other side effects in patients who received ceftazidime-avibactam plus metronidazole was 4.83%, while the incidence rate in the meropenem arm was 4.98%; it was higher in the meropenem arm but not significant, as shown in Figure 5 (RR: 0.97; 95% CI: 0.79 to 1.91; p = 0.78) [29]. The overall incidence rate of all side effects in patients who received ceftazidime-avibactam plus metronidazole was 5.57%, while the incidence rate in the meropenem arm was 4.56%; it was higher but not significant in the ceftazidime-avibactam plus metronidazole arm, as shown in Figure 4 and Figure 5 (RR: 1.22; 95% CI: 0.78 to 1.93; p = 0.39).

Efficacy Profile

**Figure 6:**
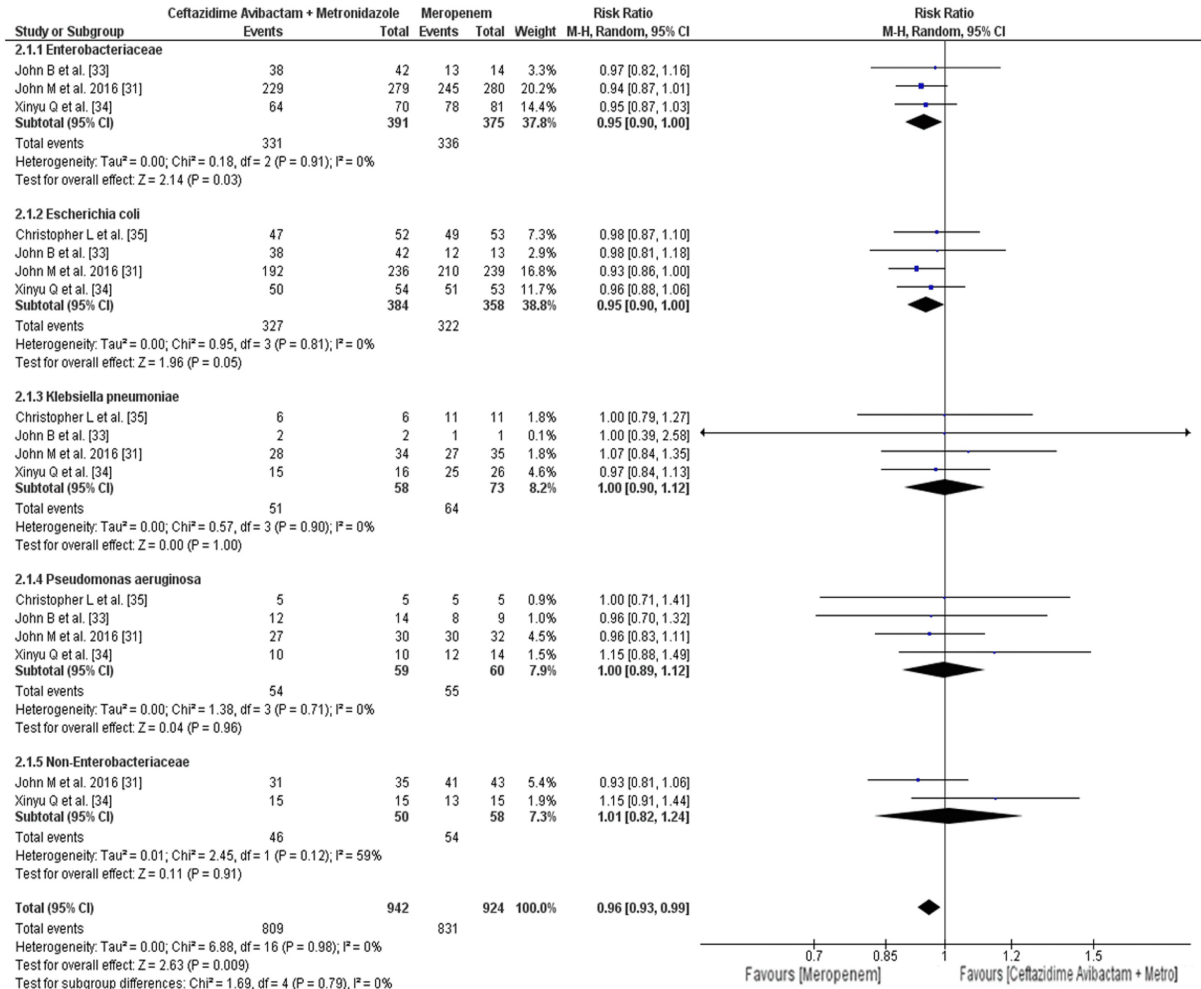
Clinical responses at the test-of-cure visits for patients with ceftazidime-susceptible Gram-negative pathogens The horizontal line represents 95% CI; a vertical line represents a “no difference” point between the two groups; squares are risk ratios; diamonds are pooled risk ratios CI: confidence interval; MH: Mantel-Haenszel; Metro: metronidazole

The assessment of the efficacy profile concentrated on the clinical response at the test of cure visit (Figure 6). The rate of clinical response in patients treated with with ceftazidime-avibactam plus metronidazole who had Enterobacteriaceae with ceftazidime susceptibility was 84.65%, while the rate in the meropenem arm was 89.6%; it was higher in the meropenem arm but not significant (RR: 0.95; 95% CI: 0.90 to 1.00; p = 0.91). The rate of clinical response in patients treated with with ceftazidime-avibactam plus metronidazole who had Escherichia coli with ceftazidime susceptibility was 85.15%, while the rate in the meropenem arm was 89.94%; it was higher in the meropenem arm (RR: 0.95; 95% CI: 0.90 to 1.00; p = 0.05). The rate of clinical response in patients treated with with ceftazidime-avibactam plus metronidazole who had Klebsiella pneumoniae with ceftazidime susceptibility was 87.93%, while the rate in the meropenem arm was 87.67%; it was higher in the ceftazidime-avibactam plus metronidazole arm but not significant (RR: 1.00; 95% CI: 0.90 to 1.12; p = 1.00). The rate of clinical response in patients treated with with ceftazidime-avibactam plus metronidazole who had Pseudomonas aeruginosa with ceftazidime susceptibility was 91.52%, while the rate in the meropenem arm was 91.66%; it was higher in the meropenem arm but not significant (RR: 1.00; 95% CI: 0.89 to 1.12; p = 0.96). The rate of clinical response in patients treated with with ceftazidime-avibactam plus metronidazole who had non-Enterobacteriaceae with ceftazidime susceptibility was 92%, while the rate in the meropenem arm was 93.10%; it was higher in the meropenem arm but not significant: (RR: 1.01; 95% CI: 0.82 to 1.24; P = 0.91).

The overall rate of clinical response in patients treated with with ceftazidime-avibactam plus metronidazole with ceftazidime-susceptible organism was 85.88%, while the rate in the meropenem arm was 89.93%; it was significant higher in the meropenem arm, as shown in Figure 6 (RR: 0.96; 95% CI: 0.93 to 0.99; p = 0.009) [29].

**Figure 7:**
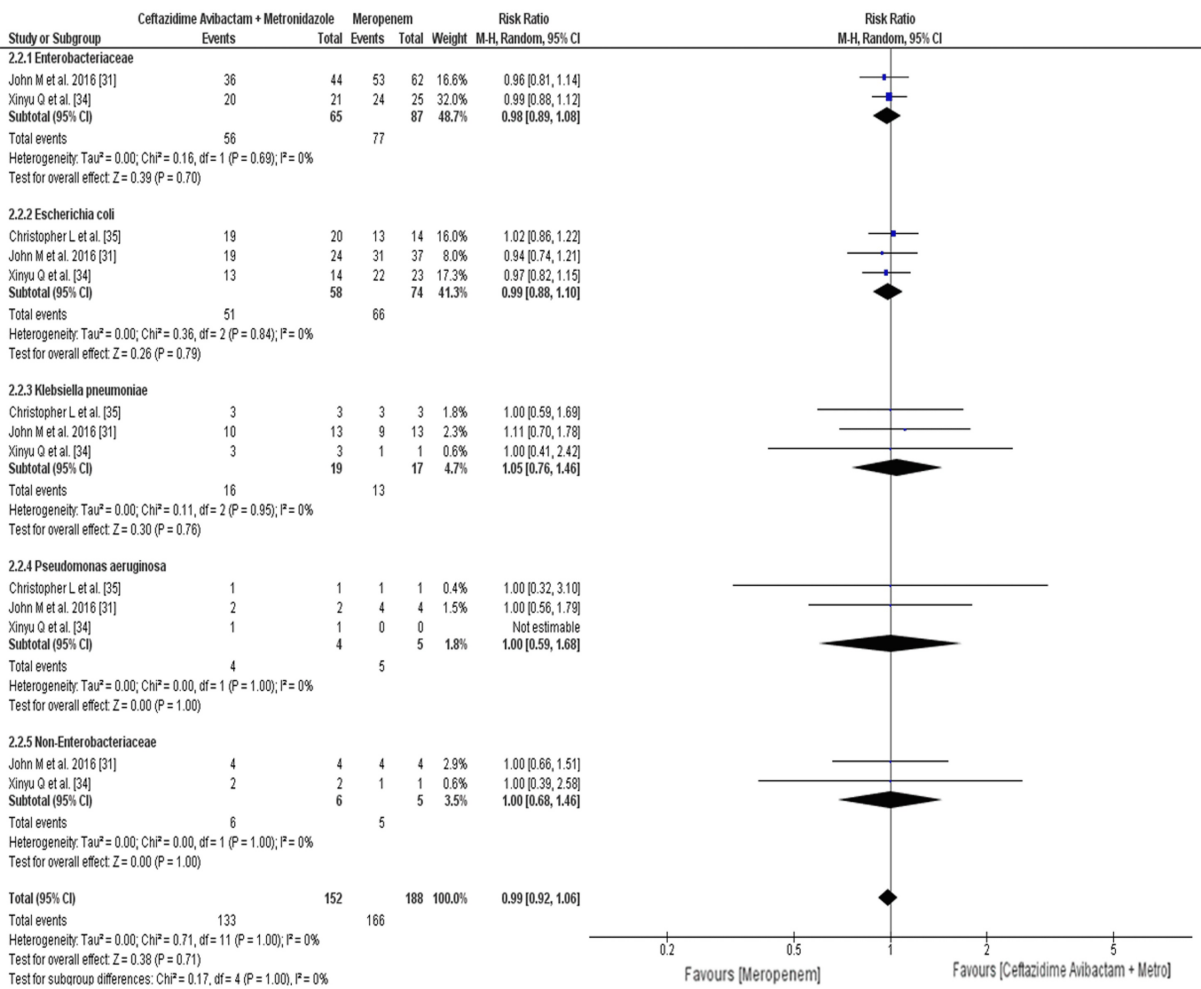
Clinical responses at the test-of-cure visits for patients with ceftazidime-resistant Gram-negative pathogens The horizontal line represents 95% CI; a vertical line represents a “no difference” point between the two groups; squares are risk ratios; diamonds are pooled risk ratios CI: confidence interval; MH: Mantel-Haenszel; Metro: metronidazole

The rate of clinical response in patients treated with with ceftazidime-avibactam plus metronidazole who had Enterobacteriaceae with ceftazidime resistance was 86.15%, while the rate in the meropenem arm was 88.50%; it was higher in the meropenem arm but not significant (RR: 0.98; 95% CI: 0.89 to 1.08; p = 0.70). The rate of clinical response in patients treated with with ceftazidime-avibactam plus metronidazole who had Escherichia coli with ceftazidime resistance was 87.93%, while the rate in the meropenem arm was 89.18%; it was higher in the meropenem arm but not significant (RR: 0.99; 95% CI: 0.88 to 1.10; p = 0.79). The rate of clinical response in patients treated with with ceftazidime-avibactam plus metronidazole who had Klebsiella pneumoniae with ceftazidime resistance was 84.21%, while the rate in the meropenem arm was 76.47%; it was higher in the ceftazidime-avibactam plus metronidazole arm but not significant (RR: 1.05; 95% CI,:0.76 to 1.46; p = 0.76). The rate of clinical response in patients treated with ceftazidime-avibactam plus metronidazole who had Pseudomonas aeruginosa with ceftazidime resistance was 100%, while the rate in the meropenem arm was 100% (RR: 1.00; 95% CI: 0.59 to 1.68; p = 1.00). The rate of clinical response in patients treated with ceftazidime-avibactam plus metronidazole who had non-Enterobacteriaceae with ceftazidime resistance was 100%, while the rate in the meropenem arm was 100% (RR: 1.00; 95% CI: 0.68 to 1.46; p = 1.00) (Figure 7). 

The overall rate of clinical response in patients treated with with ceftazidime-avibactam plus metronidazole with ceftazidime-resistance organism was 87.5%, while the rate in the meropenem arm was 88.29%; it was higher in the meropenem arm but not significant, as shown in Figure 7 (RR: 0.99; 95% CI: 0.92 to 1.06; p = 0.71) [29].

Sensitivity Analysis

We conducted a sensitivity analysis using a jackknife technique, in which each study was eliminated separately to examine the robustness of the pooled RR with 95% CI (Tables 3-4). The diversity in heterogeneity in the research might be attributed to differences in side effect manifestation and treatment duration.

**Table 3:** Sensitivity analysis of side effects CI: confidence interval; RR: risk ratio

Side effects	RR	95% CI	P	I^2^
Nausea				
Mazuski et al., 2016 [31]	1.90	[0.85, 4.27]	0.12	60%
Rodgers et al., 2022 [32]	2.00	[1.08, 3.73]	0.03	43%
Qin et al., 2017 [34]	1.40	[0.97, 2.02]	0.08	0%
Lucasti et al., 2013 [35]	1.74	[0.92, 3.30]	0.09	59%
Vomiting				
Mazuski et al., 2016 [31]	2.42	[1.30, 4.51]	0.006	0%
Rodgers et al., 2022 [32]	2.17	[1.32, 3.59]	0.002	0%
Bradley et al., 2019 [33]	2.53	[1.53, 4.17]	0.0003	0%
Qin et al., 2017 [34]	2.66	[1.60, 4.42]	0.0002	0%
Lucasti et al., 2013 [35]	2.30	[1.43, 3.94]	0.003	0%
Diarrhea				
Mazuski et al., 2016 [31]	1.07	[0.51, 2.24]	0.86	25%
Rodgers et al., 2022 [32]	1.43	[0.51, 3.99]	0.50	81%
Qin et al., 2017 [34]	2.25	[1.36, 3.72]	0.002	0%
Constipation				
Mazuski et al., 2016 [31]	0.83	[0.27, 2.52]	0.74	46%
Rodgers et al., 2022 [32]	0.73	[0.18, 2.93]	0.65	67%
Qin et al., 2017 [34]	0.45	[0.25, 0.83]	0.01	0%
Pyrexia				
Mazuski et al., 2016 [31]	0.98	[0.63, 1.52]	0.92	0%
Rodgers et al., 2022 [32]	0.89	[0.60, 1.33]	0.57	0%
Bradley et al., 2019 [33]	0.98	[0.69, 1.39]	0.90	0%
Qin et al., 2017 [34]	1.06	[0.72, 1.55]	0.77	0%
Lucasti et al., 2013 [35]	1.02	[0.70, 1.50]	0.91	0%
Respiratory disorders				
Mazuski et al., 2016 [31]	0.80	[0.50, 1.30]	0.37	0%
Rodgers et al., 2022 [32]	0.86	[0.53, 1.38]	0.52	0%
Bradley et al., 2019 [33]	0.85	[0.56, 1.30]	0.46	0%
Qin et al., 2017 [34]	0.81	[0.49, 1.34]	0.41	0%
Lucasti et al., 2013 [35]	0.75	[0.49, 1.16]	0.20	0%
Renal disorders				
Mazuski et al., 2016 [31]	0.92	[0.43, 1.96]	0.83	0%
Rodgers et al., 2022 [32]	1.56	[0.56, 4.32]	0.39	27%
Bradley et al., 2019 [33]	1.35	[0.58, 3.10]	0.49	32%
Qin et al., 2017 [34]	1.41	[0.61, 3.27]	0.42	32%
Lucasti et al., 2013 [35]	1.76	[0.83, 3.76]	0.14	0%
Nervous venous disorder				
Mazuski et al., 2016 [31]	1.21	[0.57, 2.54]	0.62	0%
Rodgers et al., 2022 [32]	0.96	[0.66, 1.40]	0.84	0%
Qin et al., 2017 [34]	0.97	[0.67, 1.41]	0.87	0%

**Table 4:** Sensitivity analysis of clinical response at the test of cure visit CI: confidence interval; RR: risk ratio

Clinical response	RR	95% CI	P	I^2^
Enterobacteriaceae with ceftazidime susceptibility				
Mazuski et al., 2016 [31]	0.95	[0.88, 1.03]	0.22	0%
Bradley et al., 2019 [33]	0.94	[0.89, 0.99]	0.03	0%
Qin et al., 2017 [34]	0.94	[0.88, 1.01]	0.08	0%
Escherichia coli with ceftazidime susceptibility				
Mazuski et al., 2016 [31]	0.97	[0.91, 1.04]	0.37	0%
Bradley et al., 2019 [33]	0.95	[0.90, 1.00]	0.05	0%
Qin et al., 2017 [34]	0.95	[0.89, 1.00]	0.07	0%
Lucasti et al., 2013 [35]	0.94	[0.89, 1.00]	0.05	0%
Klebsiella pneumoniae with ceftazidime susceptibility				
Mazuski et al., 2016 [31]	0.98	[0.87, 1.11]	0.78	0%
Bradley et al., 2019 [33]	1.00	[0.89, 1.12]	1.00	0%
Qin et al., 2017 [34]	1.03	[0.87, 1.22]	0.71	0%
Lucasti et al., 2013 [35]	1.00	[0.88, 1.13]	1.00	0%
Pseudomonas aeruginosa with ceftazidime susceptibility				
Mazuski et al., 2016 [31]	1.05	[0.88, 1.25]	0.58	0%
Bradley et al., 2019 [33]	1.00	[0.89, 1.13]	0.97	0%
Qin et al., 2017 [34]	0.97	[0.85, 1.09]	0.59	0%
Lucasti et al., 2013 [35]	1.00	[0.88, 1.12]	0.96	0%
Non-Enterobacteriaceae with ceftazidime susceptibility				
Mazuski et al., 2016 [31]	1.15	[0.91, 1.44]	0.24	N/A
Qin et al., 2017 [34]	0.93	[0.81, 1.06	0.29	N/A
Enterobacteriaceae with ceftazidime resistance				
Mazuski et al., 2016 [31]	0.99	[0.88, 1.12]	0.90	N/A
Qin et al., 2017 [34]	0.96	[0.81, 1.14]	0.62	N/A
Escherichia coli with ceftazidime resistance				
Mazuski et al., 2016 [31]	1.00	[0.88, 1.12]	0.94	0%
Qin et al., 2017 [34]	1.00	[0.86, 1.15]	0.96	0%
Lucasti et al., 2013 [35]	0.96	[0.84, 1.11]	0.59	0%
Klebsiella pneumoniae with ceftazidime resistance				
Mazuski et al., 2016 [31]	1.00	[0.64, 1.57]	1.00	0%
Qin et al., 2017 [34]	1.06	[0.75, 1.50]	0.74	0%
Lucasti et al., 2013 [35]	1.09	[0.72, 1.64]	0.70	0%
Pseudomonas aeruginosa with ceftazidime resistance				
Mazuski et al., 2016 [31]	1.00	[0.32, 3.10]	1.00	N/A
Qin et al., 2017 [34]	1.00	[0.59, 1.68]	1.00	0
Lucasti et al., 2013 [35]	1.00	[0.56, 1.79]	1.00	N/A
Non-Enterobacteriaceae with ceftazidime resistance				
Mazuski et al., 2016 [31]	1.00	[0.39, 2.58]	1.00	N/A
Qin et al., 2017 [34]	1.00	[0.66, 1.51]	1.00	N/A

Discussion

Intra-abdominal infections cover a range of medical conditions that vary in complexity, primarily categorized as uncomplicated and complicated infections. Uncomplicated intra-abdominal infections, such as acute appendicitis and cholecystitis, affect only one organ and do not extend into the peritoneum. In contrast, complicated infections involve the spread of the infection into the peritoneal cavity, requiring more intensive treatment approaches. The prognosis for these infections is influenced by factors such as the infection's origin, the patient's overall health, and previous treatments. Recent advancements in antibiotics and imaging techniques have significantly improved patient outcomes in these cases [1-4].

The pathogens responsible for intra-abdominal infections primarily include Gram-positive cocci and Gram-negative Enterobacteriaceae, as well as obligate anaerobes. Common isolates found in these infections include Escherichia coli and Klebsiella pneumoniae, which necessitate the use of broad-spectrum antibiotics for effective empirical treatment. Among the treatment options, meropenem stands out as a carbapenem antibiotic known for its low toxicity and broad spectrum of activity. Additionally, ceftazidime-avibactam and metronidazole are also important in managing these infections [5-8].

Meropenem, as a β-lactam antibiotic in the carbapenem class, is frequently employed for severe and nosocomial infections. Its low toxicity and broad mode of action effectively prevent bacterial cell wall formation, contributing to its effectiveness. Notably, meropenem exhibits time-dependent bactericidal activity, ensuring that its levels remain above the minimum inhibitory concentration for prolonged periods, which is crucial for achieving therapeutic success. This broad-spectrum effectiveness solidifies meropenem's role as a standard treatment choice for various intra-abdominal infections, highlighting its importance in the ever-evolving landscape of antibiotic therapy for serious infections [9-13].

In treating intra-abdominal infections, the combination of ceftazidime-avibactam and metronidazole offers a promising therapeutic option. Ceftazidime, a third-generation cephalosporin, is particularly effective against Pseudomonas aeruginosa, while avibactam acts as a β-lactamase inhibitor, enhancing the efficacy of ceftazidime against resistant strains. This combination targets over 99% of Enterobacteriaceae and 95% of Pseudomonas aeruginosa isolates. Metronidazole complements this therapy by effectively disrupting the DNA of anaerobic bacteria. Together, these medications improve the likelihood of successful treatment outcomes, especially in complicated infections with resistant organisms [14-21].

When comparing the safety of ceftazidime-avibactam plus metronidazole compared to meropenem, it was found that the overall rate of adverse effects was slightly higher in the ceftazidime-avibactam group, at 5.57%, compared to 4.56% in the meropenem arm. Although this suggests a greater incidence of side effects with the combination therapy, the difference was not statistically significant (RR, 1.22; 95% CI, 0.78 to 1.93; P = 0.39).

Looking at efficacy, the clinical response rate in patients treated with ceftazidime-avibactam and metronidazole for ceftazidime-susceptible organisms was 85.88%. In contrast, the response rate in the meropenem group was 89.93%. This difference is statistically significant, indicating that meropenem had a better response rate (RR, 0.96; 95% CI, 0.93 to 0.99; P = 0.009). For patients with ceftazidime-resistant organisms, the response rate for ceftazidime-avibactam was 87.5%, while meropenem achieved a slightly higher rate of 88.29%, though this difference was not statistically significant (RR, 0.99; 95% CI, 0.92 to 1.06; P = 0.71).

The sensitivity analysis evaluated the RR, CI, p-values, and I² for various side effects across multiple studies. Nausea showed RR values ranging from 1.40 to 2.00, with Rodgers et al. [32] (RR: 2.00, p = 0.03) demonstrating significant findings and moderate heterogeneity (I² = 43-60%). Vomiting consistently showed significant increases, with RR values between 2.17 and 2.66, particularly highlighted by Qin et al. [34] (p = 0.0002). Diarrhea results were mixed; Mazuski et al. [31] reported a non-significant RR of 1.07, whereas Qin et al. [34] indicated a significant increase (RR: 2.25, p = 0.002). Constipation was associated with decreased incidence, especially in Qin et al. [34] (RR: 0.45, p = 0.01). Pyrexia and respiratory disorders showed no significant differences, with RR values near 1. Renal disorders also had no significant findings, while nervous venous disorders indicated no substantial increase in incidence. Overall, the analysis highlights significant concerns regarding nausea and vomiting, while other side effects exhibited lower incidences, necessitating careful interpretation and further investigation in clinical settings.

The sensitivity analysis assessed the clinical response at the Test-of-Cure visit for various bacterial strains in relation to ceftazidime susceptibility and resistance. For Enterobacteriaceae with ceftazidime susceptibility, RR were close to 1, with Bradley et al. [33] (RR: 0.94, p = 0.03) showing significant results. Escherichia coli and Klebsiella pneumoniae similarly demonstrated RR values near 1, indicating no significant differences in clinical response. In contrast, Pseudomonas aeruginosa had RR values ranging from 0.97 to 1.05, suggesting comparable effectiveness. Non-Enterobacteriaceae and resistant strains exhibited RR values around 1, indicating similar clinical outcomes regardless of susceptibility status. Overall, the analysis highlights that ceftazidime maintains an effective clinical response across various bacterial strains, with most studies showing no significant differences in treatment outcomes.

In summary, while the combination of ceftazidime-avibactam plus metronidazole shows potential, especially against resistant organisms, meropenem consistently demonstrated higher efficacy in treating ceftazidime-susceptible infections. This reinforces meropenem's position as a strong option for managing intra-abdominal infections.

## Conclusions

This systematic review and meta-analysis demonstrate that ceftazidime-avibactam combined with metronidazole is a viable alternative to meropenem for treating intra-abdominal infections. While meropenem showed slightly higher clinical efficacy and lower gastrointestinal side effects, the combination therapy offers a comparable safety profile, with an overall incidence rate of side effects at 5.57% for ceftazidime-avibactam plus metronidazole compared to 4.56% for meropenem. The clinical response rate for ceftazidime-resistant organisms was 87.5% for the combination therapy versus 88.29% for meropenem, while for ceftazidime-susceptible organisms, it was 85.88% versus 89.93%, indicating a significant difference. Further research is warranted to explore its potential in clinical practice, particularly in settings with rising antibiotic resistance. Overall, this review contributes valuable insights into optimizing treatment strategies for intra-abdominal infections.
